# Pericytes: multitasking cells in the regeneration of injured, diseased, and aged skeletal muscle

**DOI:** 10.3389/fnagi.2014.00245

**Published:** 2014-09-18

**Authors:** Alexander Birbrair, Tan Zhang, Zhong-Min Wang, Maria L. Messi, Akiva Mintz, Osvaldo Delbono

**Affiliations:** ^1^Department of Internal Medicine-Gerontology, Wake Forest School of MedicineWinston-Salem, NC, USA; ^2^Neuroscience Program, Wake Forest School of MedicineWinston-Salem, NC, USA; ^3^Department of Neurosurgery, Wake Forest School of MedicineWinston-Salem, NC, USA

**Keywords:** pericytes, skeletal muscle, regeneration, innervation, fat formation, fibrous tissue, angiogenesis, aging

## Abstract

Pericytes are perivascular cells that envelop and make intimate connections with adjacent capillary endothelial cells. Recent studies show that they may have a profound impact in skeletal muscle regeneration, innervation, vessel formation, fibrosis, fat accumulation, and ectopic bone formation throughout life. In this review, we summarize and evaluate recent advances in our understanding of pericytes' influence on adult skeletal muscle pathophysiology. We also discuss how further elucidating their biology may offer new approaches to the treatment of conditions characterized by muscle wasting.

## Introduction

In skeletal muscle, small blood vessels called capillaries surround each myofiber (Poole et al., 2008). The capillary wall consists of endothelial cells and pericytes. The latter are wrapped by the capillary basal lamina, located on the abluminal surface of the endothelial capillary tube. The word *pericyte* derives from the Greek *kytos*, a hollow vessel, appropriately describing a cell surrounding a blood vessel.

Electron microscopy studies estimated the pericyte-to-endothelial-cell ratio for the overall coverage of microvessels in striated skeletal muscle as approximately 1:100 (Shepro and Morel, 1993). However, the analyzed samples were unstained, so the technique could not precisely distinguish pericytes from other surrounding cells. Pericytes are defined by their anatomical location in combination with several molecular markers (Kunz et al., 1994; Verbeek et al., 1994; Lindahl et al., 1997; Ozerdem et al., 2001), and the great advances in fluorescent imaging techniques suggest reanalyzing this ratio.

In most peripheral organs, pericytes are derived from the mesoderm (Armulik et al., 2011), but their origin in skeletal muscle has not been explored sufficiently. Specific pericyte subpopulations with distinct roles in the skeletal muscle have been described (Birbrair et al., 2013c). Whether pericyte subsets derive from different embryonic tissues remains unknown. CNS pericytes derive from the ectoderm (Bergwerff et al., 1998; Etchevers et al., 2001; Korn et al., 2002; Heglind et al., 2005). Brain pericytes exhibit both exclusive markers (Bondjers et al., 2006) and markers shared with skeletal muscle pericytes (Armulik et al., 2011). Whether and how pericyte functions in skeletal muscle differ from those in the brain remains to be explored. Brain and skeletal muscle pericytes express NG2 proteoglycan (Armulik et al., 2011; Birbrair et al., 2013c) and react to injury by forming fibrotic scars (Popa-Wagner et al., 2006; Dulauroy et al., 2012), suggesting that, independent of location, they share some properties.

**Figure 1:**
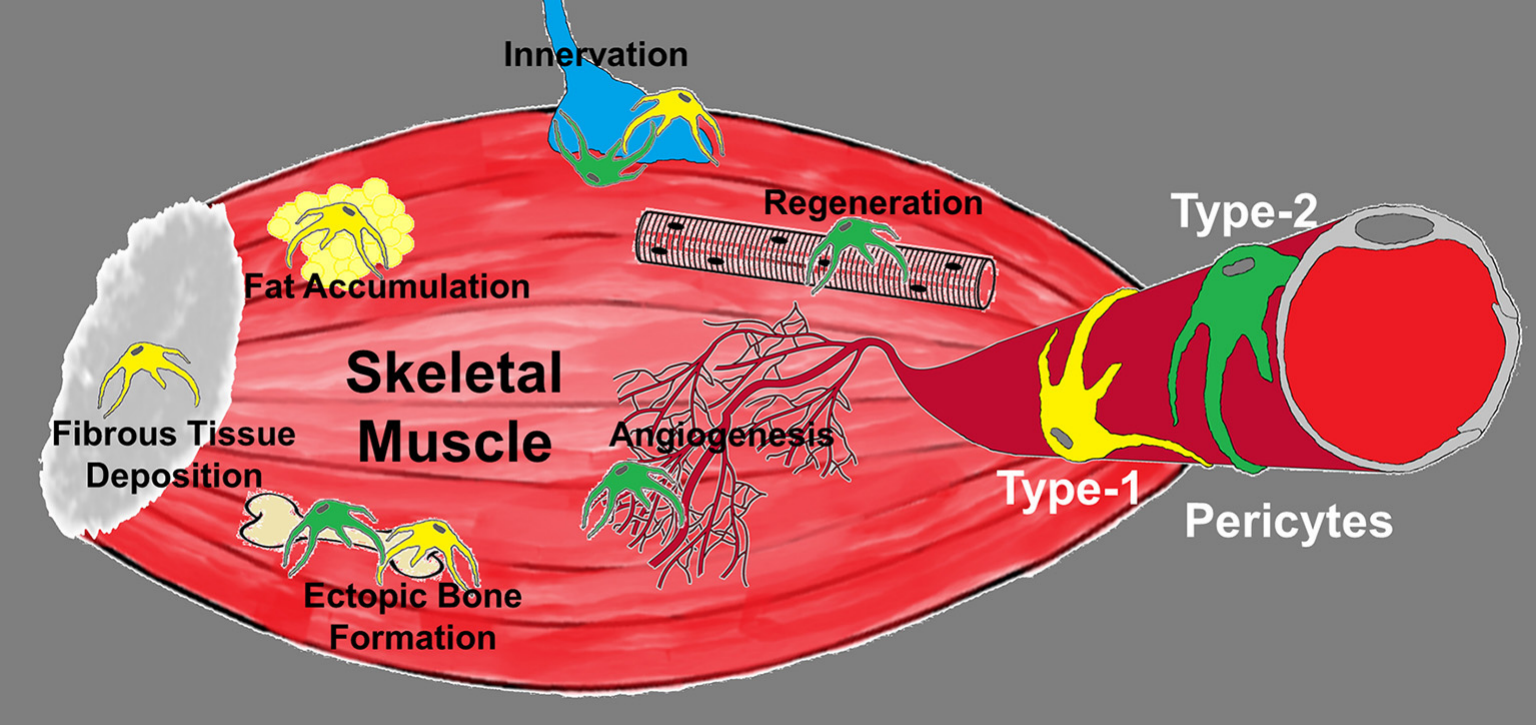
**Diagram depicting the roles of pericyte subtypes in skeletal muscle**. Type-1 (yellow) and type-2 (green) pericytes are associated with blood vessels and differentially committed to tissue formation. Type-1 pericytes form adipose and fibrous tissue, while type-2 pericytes cooperate with myogenesis and angiogenesis. Their role in muscle innervation and ectopic bone deposition remains unexplored.

Transplanting fluorescently marked embryonic tissues (e.g., mesoderm, endoderm, ectoderm, neural crest cells) into unmarked embryonic skeletal muscle may provide some clues on pericyte ancestors. Another approach would use transgenic mice for genetic tracking of cells from different embryonic tissues to pinpoint the origin of skeletal muscle pericytes and whether it differs for the recently identified subpopulations (Figure 1) (Birbrair et al., 2013a,c,d, 2014, 2015).

Pericytes are heterogeneous, exhibiting major differences depending on the tissue from which they have been isolated (Sims, 2000; Bondjers et al., 2006). Their functions seem to be tissue-specific, and very little is known about their function in skeletal muscle. Here, we present an overview of the current knowledge on their participation in adult muscle regeneration, reinnervation, vascularization, fibrosis, fat formation, and calcium deposition.

## Pericyte participation in skeletal muscle regeneration

Skeletal muscle comprises about 40% of the human body by weight. It is a highly organized network of different types of cells, neurovascular structures, and connective tissue. It may undergo physiological changes based on everyday physical activity, and due to its superficial location, it is constantly subjected to different grades of traumatic injury. Nevertheless, in young healthy subjects, skeletal muscle is well recognized for its remarkably robust endogenous capacity to repair itself (Huard et al., 2002; Cossu and Biressi, 2005; Relaix and Zammit, 2012). Functional loss of skeletal muscle mass and strength is observed with aging in a process known as sarcopenia (Delbono, 2011); as a secondary effect, known as cachexia, in some cancer patients; and due to trauma, vascular injuries, or degenerative muscle disorders, such as muscular dystrophy (Janssen et al., 2002; Acharyya et al., 2005; Glass, 2010; Vilquin et al., 2011). Muscle wasting can cause severe debilitating weakness and represents a significant clinical problem with few solutions (Vilquin et al., 2011). All of these conditions would benefit from approaches that promote skeletal muscle regeneration, but they would require complete understanding of the complexity of the cellular mechanisms involved.

The most well-studied myogenic cells in skeletal muscle are the muscle-specific stem cells called *satellite cells*, which are located between the basal lamina and sarcolemma of individual myofibers (Charge and Rudnicki, 2004; Zammit et al., 2006; Boldrin and Morgan, 2013). These cells participate in skeletal muscle repair in response to injury, but they are scarce and difficult to isolate (Berardi et al., 2014). Other problems include poor survival, incompatibility with systemic delivery, and host rejection (Giordano and Galderisi, 2010). Hence, researchers have explored and identified other skeletal muscle cells with high myogenic potential. They include muscle-derived stem cells (MDSCs; Lee et al., 2000; Qu-Petersen et al., 2002; Lavasani et al., 2006; Urish et al., 2009; Drowley et al., 2010); CD133+ progenitor cells (Torrente et al., 2004; Peault et al., 2007; Negroni et al., 2009); endothelial cells (Zheng et al., 2007); PW1+ interstitial cells (Mitchell et al., 2010; Pannerec et al., 2013); muscle side population (SP) cells (Gussoni et al., 1999; Asakura and Rudnicki, 2002; Bachrach et al., 2004); and pericytes (Dellavalle et al., 2007, 2011; Birbrair et al., 2013a,d).

Note that, besides cell therapy, all these cells may contribute to endogenous skeletal muscle formation, defined as the creation of new myofibers by proliferation, recruitment, migration and fusion of mononucleated cells (Yin et al., 2013).

The exact relationship between the different skeletal muscle cell types with myogenic potential and whether they all derive from one source have yet to be established. Future studies must define their specific contributions to muscle formation. One approach might be to use mouse models in which only one of these cell populations is ablated. Such a study showed that satellite cells are essential for skeletal muscle regeneration (Lepper et al., 2011; McCarthy et al., 2011; Murphy et al., 2011; Sambasivan et al., 2011). However, efficient skeletal muscle formation also depends on the interaction of cell types without myogenic potential, such as connective tissue fibroblasts (Barnes and Glass, 2011). In future studies ablating each of these cell populations separately, followed by skeletal muscle regeneration analysis, we may learn that they interact physically and release factors directly affecting each other's myogenic capacity.

Pericytes are myogenic *in vitro* (Dellavalle et al., 2007). When injected into injured skeletal muscle, they induce a higher muscle regenerative index than enriched myoblasts (Crisan et al., 2008b). Intra-arterial transplantation in animal models of muscular dystrophies demonstrated that they largely engrafted and may improve skeletal muscle function (Sampaolesi et al., 2003, 2006). Tedesco et al. (2012) found that transplantation of genetically modified pericytes resulted in functional amelioration of the dystrophic phenotype.

A more interesting question is whether endogenous pericytes contribute to skeletal muscle formation. Fate-tracking experiments with alkaline phosphatase (AP) Cre-ER mice proved that pericytes associated with blood vessels contribute to postnatal skeletal muscle growth by type; for example, less in tibialis anterior than diaphragm. They can assume satellite cells' position and become satellite cells (Dellavalle et al., 2011). Through unknown mechanisms, pericytes' contribution increases greatly during skeletal muscle regeneration in response to chemical injury (Dellavalle et al., 2011). Whether pericytes expand in the skeletal muscle following physical injury (for instance, in response to exercise) remains unknown; and, if so, whether the mechanisms are similar to those activated in response to chemical injury must be addressed (Boppart et al., 2013).

The molecular mechanisms activating and orchestrating pericytes' transition from quiescence to regenerating capacity in the skeletal muscle are also unknown. Again, whether selective ablation of pericytes from skeletal muscle will prevent or otherwise affect regeneration will clarify whether they can be replaced by other cell types with myogenic capacity. We propose that due to their ability to secrete several growth factors, pericytes may be required to induce other cell types to adopt a myogenic fate (Sato and Rifkin, 1989; Shepro and Morel, 1993; Davis et al., 1996; Yamagishi et al., 1999; Brown et al., 2001; Reinmuth et al., 2001; Hirschi et al., 2003; Niimi, 2003; Armulik et al., 2005; Paquet-Fifield et al., 2009; Shimizu et al., 2011). A global analysis of candidate growth factors secreted by skeletal muscle pericytes that promote skeletal muscle regeneration is required.

Our recent work reported the presence of two pericyte subpopulations in the skeletal muscle. Type-1 (Nestin-GFP-/NG2-DsRed+) and type-2 (Nestin-GFP+/NG2-DsRed+) pericytes are in close proximity to blood vessel endothelial cells and co-localize with other pericytic markers (Birbrair et al., 2013c), but only type-2 is myogenic and participates in skeletal muscle regeneration (Birbrair et al., 2013a). Type-1 may not express the specific receptors needed to mediate the signaling pathways required for myogenic differentiation. Future studies should reveal the specific signaling pathways and why only one subpopulation can be induced to a myogenic fate. We also found that only type-2 pericytes can enter the satellite cell compartment and express satellite cell marker Pax7 (Birbrair et al., 2013d).

The host microenvironment is critical for their myogenicity; for example, in older mice, the muscular regenerative capacity of type-2 pericytes is limited (Birbrair et al., 2013d), suggesting that it might be improved by modifying the deleterious aged muscle microenvironment. Approaches aimed at changing the old skeletal muscle environment have been reported. For instance, 3D hydrogel was used to rejuvenate pericytes derived from aged skeletal muscle, and their myogenic capacity improved (Fuoco et al., 2014).

To what extent impaired type-2 myogenicity leads to myofiber loss or skeletal muscle atrophy as compared to the effects of other myogenic cells in the skeletal muscle has yet to be defined. No one has yet studied whether intrinsic pericyte changes may impair skeletal muscle regeneration with aging, as recently reported in satellite cells (Bentzinger and Rudnicki, 2014; Bernet et al., 2014; Cosgrove et al., 2014; Sousa-Victor et al., 2014). Further, are pericyte autonomous changes with aging reversible? Is one pericyte subtype more prone to senescence or apoptosis? Does the aging environment select for a pericyte subtype with poorer myogenic potential? Does the number of distinct pericyte subpopulations change with aging and in diseased dystrophic skeletal muscle?

The only marker differentially expressed in skeletal muscle pericytes is Nestin-GFP, which is also expressed in satellite cells (Birbrair et al., 2011). Thus, only the combination of Nestin-GFP and NG2-DsRed expression distinguishes the pericyte subpopulations reported in the muscle interstitium (Birbrair et al., 2013c). Future studies to determine whether the absence of endogenous type-2 pericytes compromises skeletal muscle regeneration, as with satellite cells (Barnes and Glass, 2011; Dellavalle et al., 2011; Lepper et al., 2011; McCarthy et al., 2011), will require discovery of novel markers selectively expressed in a pericyte subpopulation. To define them, investigators must first characterize the expression profiles of type-1 and type-2 pericytes. Better characterization may also advance therapies that target specific receptors. For instance, β-agonist therapy has shown potential in skeletal muscle repair (Beitzel et al., 2004); whether skeletal muscle pericytes express beta adrenergic receptors in response to these drugs is not known, although pericytes from other tissues do so (Kelley et al., 1988; Elfont et al., 1989; Ferrari-Dileo et al., 1992; Zschauer et al., 1996; Rucker et al., 2000; Quignard et al., 2003; Mendez-Ferrer et al., 2010a,b; Daly and McGrath, 2011; Lee et al., 2014).

Recent studies demonstrate that pericytes are involved in skeletal muscle regeneration as described above (Crisan et al., 2008b; Dellavalle et al., 2011; Birbrair et al., 2013a). However, the molecular mechanisms underlying the recruitment of pericytes in this complex process are unknown.

## Pericytes in skeletal muscle reinnervation

Muscle innervation is essential for maintaining mass and function with aging (Delbono, 2003, 2011). Denervation can result from trauma, immobility, unloading, infections, vasculitis, neuropathies, autoimmune processes, neoplasms, amyotrophic lateral sclerosis, and aging (Polak et al., 1988; Nikawa et al., 2004; Argiles et al., 2006; Glass, 2010; Bonaldo and Sandri, 2013). Loss of the nerve supply causes robust progressive skeletal muscle degeneration (Nikawa et al., 2004; Batt et al., 2006; Aagaard et al., 2010; Ohlendieck, 2011). Focal injuries to the peripheral nerves are often followed by complete recovery due to the capacity of peripheral axons to reoccupy neuromuscular junctions on denervated muscle fibers (Young, 1974; Rich and Lichtman, 1989; Nguyen et al., 2002). Even without nerve injury, the nerve terminal is constantly remodeled during degeneration and regeneration of skeletal muscle fibers, as this neuromuscular connection is needed for functional recovery of skeletal muscle (Li and Thompson, 2011). Rounds of denervation and reinnervation are evidenced by the random distribution of myofiber types across the skeletal muscle and their increased clustering with age (Larsson, 1995; Andersen, 2003; Rowan et al., 2012). Over the years, complete functional recovery is significantly reduced (Verdu et al., 2000; Kawabuchi et al., 2011). Whether these changes are controlled by the muscular, neural, or both systems is not known. Myofiber denervation has been demonstrated in mice and elderly humans (Hashizume et al., 1988; Kanda and Hashizume, 1989, 1992; Einsiedel and Luff, 1992; Doherty et al., 1993; Johnson et al., 1995; Zhang et al., 1996; Delbono, 2003; Aagaard et al., 2010; Valdez et al., 2010; Chai et al., 2011).

Regenerating axons grow through a complex microenvironment during the reinnervation process (Rich and Lichtman, 1989). They probably recognize and attach to postsynaptic sites guided by non-synaptic muscle fiber membranes and the membranes of glial cell processes (Kang et al., 2007). Schwann cells are widely believed to support axonal growth and to provide important cues that enable nerve fibers to reach the vacant synaptic sites (Chen et al., 2007). They also provide such important growth factors as neurotrophins, which ensure correct reinnervation after experimental nerve transection (Sendtner et al., 1992; Funakoshi et al., 1993; Friedman et al., 1996; Frostick et al., 1998; Brushart et al., 2013). Regenerating axons are guided by processes extended by terminal Schwann cells at the denervated synaptic location (Kang et al., 2014). Distinct Schwann cell populations have been described, including non-myelinating, myelinating, terminal, and perisynaptic Schwann cells, and terminal Schwann-like cells of sensory neuritis (Kwan, 2013). How their roles differ in skeletal muscle reinnervation remains poorly understood. The release of neurotrophic factors may be differentially regulated in these subpopulations. Schwann cells from young and old mice have been shown to differ both morphologically and in their ability to cover the motor endplate (Chai et al., 2011). The number of cells at the neuromuscular junction increases after skeletal muscle denervation, and based on Schwann cell markers, not all are Schwann cells (Magill et al., 2007). Future studies should determine the identity of the other cells and how they contribute to reinnervation. Whether pericytes participate remains largely unexplored.

Previous studies have demonstrated that CNS perivascular pericytes can form cells that express glial markers (Dore-Duffy et al., 2006; Bonkowski et al., 2011; Jung et al., 2011; Nakagomi et al., 2011). In skeletal muscle, cells in the interstitial space differentiate into glial lineage (Romero-Ramos et al., 2002; Alessandri et al., 2004; Kondo et al., 2006; Schultz and Lucas, 2006; Arsic et al., 2008; Birbrair et al., 2011). We recently demonstrated that under optimized skeletal muscle culture conditions, only type-2 pericytes form oligodendrocyte progenitors (Birbrair et al., 2013b,c), which produce mature oligodendrocytes and Schwann cells (Zawadzka et al., 2010). Whether type-2 pericytes contribute to Schwann cell populations generally and to the newly formed Schwann cells that participate in skeletal muscle reinnervation specifically remains to be addressed.

Also, to what degree do these endogenous pericytes influence the reoccupation of synaptic sites during reinnervation? Recently, cells isolated from adult human skeletal muscle were shown to differentiate into myelinating Schwann cells and to ameliorate a critical-sized sciatic nerve injury in a murine model. Denervated skeletal muscles from treated mice exhibit substantially decreased atrophy, and the motor endplates at the postsynaptic sites reorganize (Lavasani et al., 2014). Although this study provides evidence for the therapeutic capability of skeletal muscle-derived cells *in vivo*, it did not identify these cells. As the skeletal muscle cell environment is heterogenous, future work should focus on identifying muscle-derived cells that can repair the sciatic nerve after injury. We reported that only type-2 pericytes have gliogenic potential in the skeletal muscle (Birbrair et al., 2013c), so they are immediate candidates for testing. Tracking pericyte fate by an inducible Cre system after skeletal muscle denervation will be required to address pericyte potential to form Schwann cells.

As there are several types of Schwann cells (Kwan, 2013), it would be interesting to explore whether pericytes form a specific subtype. The Wnt/beta-catenin signaling pathway is active in NG2+ cells differentiating into NG2 glia cells in the brain (White et al., 2010). As pericytes express NG2 proteoglycan and form NG2 glia cells (Birbrair et al., 2013b,c), this pathway might be activated to induce skeletal muscle reinnervation.

To understand the role of pericytes in neuromuscular junction regeneration, future studies should test the effect of ablating them after sciatic nerve injury. Mouse models using viral thymidine kinase to genetically deplete pericytes are viable and have been used recently (Cooke et al., 2012; Lebleu et al., 2013). As Schwann cells have the capacity to dedifferentiate into immature cells after sciatic nerve injury (Yang et al., 2012), do they ever become pericytes?

We envision that determining exactly what happens after denervation may provide cellular targets for pharmacological manipulation to improve skeletal muscle reinnervation. At present, the role of pericytes in this process is only speculative.

## Pericyte contribution to vessel formation in skeletal muscle

Blood supply to the skeletal muscle can be compromised after vascular injury, bone fracture, and crush injury (Blaisdell, 2002) and by complications due to such cardiovascular and metabolic diseases as atherosclerosis, heart failure, diabetes, and obesity (Baumgartner et al., 2005; Varu et al., 2010; Chi et al., 2011). In addition, the number of capillaries and arteries feeding the skeletal muscle decreases with age (Conley et al., 2000; Behnke et al., 2006), compromising its perfusion (Wahren et al., 1974; Irion et al., 1987). Compared to young mice, the capacity of old mice to form new blood vessels (angiogenesis) is impaired (Rivard et al., 1999; Shimada et al., 2004; Yu et al., 2006).

When the blood supply to tissue is partially obstructed, oxygen content decreases, leading to ischemia (Forsythe et al., 1996; Heil and Schaper, 2004). Collateral arteries and anastomoses can partially restore blood flow (Heil and Schaper, 2004), attenuating the damage caused by hypoxia, but even short-term ischemia induces necrosis, leading to inflammatory reactions. After only 5 h, an ischemic environment causes necrosis in 90 percent of skeletal muscle (Labbe et al., 1987). If revascularization fails, it can lead to limb amputation (Conrad et al., 2011). While exercise is a potent stimulus for new vessel formation in adult skeletal muscle (Booth and Thomason, 1991; Egginton, 2009), most of the conditions leading to skeletal muscle ischemia preclude exercise.

Angiogenesis is a complex process in which new blood vessels form from existing ones. It involves extensive interplay between cells and growth factors, extracellular matrix proteins, proteases, and adhesion molecules (Folkman, 1971; Caduff et al., 1986; Kilarski et al., 2009). The exact cellular mechanisms of physiological angiogenesis in skeletal muscle remain poorly understood. It requires the proliferation and migration of endothelial cells to line the interior of the blood vessels (Rousseau et al., 2000; Li et al., 2005; Lamalice et al., 2006). Macrophages and other inflammatory cells (Barbera-Guillem et al., 2002; Shireman, 2007) infiltrate the tissues after ischemia (Sica, 2010; Alexander et al., 2011) and, together with fibroblasts and myofibers (Gustafsson et al., 1999; Steinbrech et al., 1999), secrete such angiogenic molecules as vascular endothelial growth factor (VEGF), placenta growth factor (PlGF), fibroblast growth factor 2 (FGF2), and platelet-derived growth factor (PDGF; Lewis and Murdoch, 2005; Murdoch and Lewis, 2005; Murdoch et al., 2005), which are necessary to construct new blood vessels and to restore blood perfusion.

Pericytes also participate in the formation of new blood vessels (Egginton et al., 1996; Hellstrom et al., 1999; Gerhardt and Betsholtz, 2003; Bergers and Song, 2005). This participation includes phenotype changes, migration, alignment, and contacts with endothelial cells. Pericytes are the first cells to invade newly vascularized tissues (Diaz-Flores et al., 1991; Nehls et al., 1992; Reynolds et al., 2000). The hypoxic state stimulates pericyte migration and angiogenesis (Murata et al., 1994). They adopt angiogenic phenotype by shortening their processes and increasing their somatic volume (Diaz-Flores et al., 1992b, 1994b). Autoradiographic studies show that the proliferation of these activated pericytes increases (Schoefl, 1963; Cavallo et al., 1972, 1973; Sholley et al., 1977; Burger and Klintworth, 1981; Diaz-Flores et al., 1992b, 1994a,b). Pericytes guide and determine where the newly formed blood vessels spread (Nehls et al., 1992; Tsuzuki and Sasa, 1994; Ozerdem et al., 2001; Morikawa et al., 2002; Ozerdem and Stallcup, 2003) and promote endothelial cell survival (Amselgruber et al., 1999; Morikawa et al., 2002; Darland et al., 2003; Kale et al., 2005). They can form tubes (Moldovan et al., 2000; Anghelina et al., 2002, 2004, 2006; Ozerdem and Stallcup, 2003) and penetrate endothelial cells. They prevent vessel regression (Benjamin et al., 1998, 1999; Enge et al., 2002). Pericytes express PDGF receptors and respond to PDGF (Balabanov et al., 1996). Their recruitment is crucial for vessel maturation, as the lack of PDGF disrupts vessel development (Lindahl et al., 1997; Hellstrom et al., 2001). Following exercise, NF-κ B, a strong inducer of angiogenesis, is activated in a pericyte sub-population (Hyldahl et al., 2011). Under hypoxic conditions, VEGF from pericytes can stimulate other pericytes to proliferate and migrate (Yamagishi et al., 1999).

The process of angiogenesis depends on appropriate cell signaling based in the tissue microenvironment, so most of our data comes from *in vivo* studies. Dissecting and occluding the femoral artery to induce brief ischemia is a common model for studying physiological angiogenesis (Shireman and Quinones, 2005; Westvik et al., 2009).

Therapeutic angiogenesis has been pursued as a potential treatment for ischemic disorders (Isner and Asahara, 1999; Ferrara and Kerbel, 2005; Giacca and Zacchigna, 2012). Its goal is to stimulate blood vessels to grow new blood vessels (Folkman, 1995; Isner, 1996; Ferrara and Kerbel, 2005). Several cell types have been used to induce neovascularization (Kalka et al., 2000; Hamano et al., 2001; Shintani et al., 2001; Iwase et al., 2005; Swijnenburg et al., 2005). Pericyte transplantation induces angiogenesis and improves blood flow to ischemic hindlimbs in animal models (He et al., 2010; Dar et al., 2012), and based on their role in forming and stabilizing engineered blood vessels, they have been proposed for angiogenic therapy. Surprisingly, not all pericytes can induce angiogenesis. Only type-2 has angiogenic potential *in vitro*, and, *in vivo*, angiogenesis occurs when type-2, but not type-1, pericytes are injected with endothelial cells in a Matrigel plug (Birbrair et al., 2014). Type-2 can also recover blood flow in a mouse model of hindlimb ischemia (Birbrair et al., 2014), but the mechanism remains to be elucidated (Birbrair et al., 2014). Due to the short recovery time, the femoral artery is probably rebuilt by anastomoses of the proximal stump with new collateral blood vessels after intramuscular pericyte injection (Schaper and Scholz, 2003).

Pericyte subtypes in human skeletal muscle have not yet been identified and isolated, and whether their angiogenic potential differs remains unknown.

To study the physiological roles of different cell populations, genetic strategies to ablate specific cell types have been developed. Several transgenic mice now provide effective means to genetically ablate pericytes (Cooke et al., 2012; Lebleu et al., 2013), satellite cells (Dellavalle et al., 2011; Lepper et al., 2011; McCarthy et al., 2011), fibroblasts (Barnes and Glass, 2011), and macrophages (Ferenbach et al., 2012; Weisser et al., 2012) for a defined period. These studies will elucidate the exact role of each cell population in inducing and regulating skeletal muscle angiogenesis. For example, to determine whether endogenous pericytes are necessary for skeletal muscle angiogenesis after ischemia, transgenic mice with pericyte depletion, such as NG2-tk (Cooke et al., 2012; Lebleu et al., 2013), should be examined. Cell ablation studies must consider that besides pericytes, oligodendrocyte progenitors express NG2 (Encinas et al., 2011).

Understanding the molecular mechanisms of ischemia induced endogenous angiogenesis is critical. Genetically modified mice have been widely applied to study the signals required for postnatal hindlimb angiogenesis. Knockout mice allowed several groups to test whether such signals are necessary for skeletal muscle angiogenesis. Neovascularization of impaired ischemic limbs was found in mice deficient in angiotensin II type-1 receptor (Sasaki et al., 2002), endothelial-derived nitric oxide synthase (eNOS; Murohara et al., 1998), matrix metalloproteinase-9 (Johnson et al., 2004), caveolin-1 (Sonveaux et al., 2004), adiponectin (Shibata et al., 2004), PlGF (Carmeliet et al., 2001), and IL-10 (Silvestre et al., 2000). Are these molecules expressed in pericytes? The specific cell types essential to angiogenesis after hindlimb ischemia remain unclear. In addition, analysis of global knockout mutant mice is complicated by unrelated side effects in other tissues, which can be avoided only by performing conditional mutagenesis. When investigators can control the timing and location of somatic mutations in adult mice, they will be able to determine the roles of specific signaling molecules in different cell populations and the functional consequences of deleting single genes in specific cell types, such as pericytes, during skeletal muscle angiogenesis. The clinical need for interventions in ischemic illnesses leading to revascularization and the encouraging recent findings that pericytes have the potential to improve blood perfusion will stimulate these efforts.

## Pericytes in skeletal muscle fibrosis

Fibrosis is an incompletely understood process characterized by excessive accumulation of extracellular matrix components, such as collagen (Wynn, 2008). It occurs under chronic disease conditions and may affect skeletal muscle (Wynn, 2007). Fibrous tissue may prevent full functional recovery of skeletal muscle (Kasemkijwattana et al., 1998; Kaariainen et al., 2000; Huard et al., 2002; Jarvinen et al., 2005; Gharaibeh et al., 2012), which, under normal conditions, can repair itself after injury. Fibrosis directly contributes to progressive skeletal muscle dysfunction in several chronic diseases, such as Duchenne muscular dystrophy (Mann et al., 2011; Brandan and Gutierrez, 2013; Morales et al., 2013; Acuna et al., 2014); and its treatment is currently considered important for muscular dystrophies. Furthermore, one of the causes of age-related skeletal muscle stiffness, weakness, and atrophy is increased infiltration of fibrous tissue (Ryall et al., 2008; Thompson, 2009; Kragstrup et al., 2011; Walston, 2012). The regenerative potential of muscle stem cells is limited by the formation of fibrous tissue, which lacks innervation and contractile properties (Juhas and Bursac, 2013). Studies performed in mouse models clearly associate skeletal muscle fibrosis with aging (Goldspink et al., 1994; Huard et al., 2002; Jarvinen et al., 2002; Brack et al., 2007; Zhu et al., 2007; Graham et al., 2010; Trensz et al., 2010). Understanding the cellular and molecular mechanisms underlying skeletal muscle fibrosis is essential to developing effective antifibrotic therapies.

While collagen accumulation is a major feature of skeletal muscle fibrosis (Mohan and Radha, 1980; Alnaqeeb et al., 1984; Goldspink et al., 1994; Haus et al., 2007; Kragstrup et al., 2011), the source of the collagen-producing cells in various conditions is less clear. Many have been proposed, including resident fibroblasts (Thiery et al., 2009; Lieber and Ward, 2013), muscle-derived stem cells (Li and Huard, 2002), myoblasts (Li et al., 2004; Alexakis et al., 2007), endothelial cells (Zeisberg et al., 2007), pericytes (Birbrair et al., 2013d), fibroadipogenic progenitors (FAPs; Joe et al., 2010; Uezumi et al., 2011), fibrocytes (Herzog and Bucala, 2010), and even nerve-associated cells (Hinz et al., 2012). However, the exact role of these cells in skeletal muscle fibrosis is unclear, and the origin of collagen-producing cells has not been confirmed using the same methodologies (*in vitro* or *in vivo*). Furthermore, skeletal muscle fibrosis induced in different ways may recruit different cell populations. For instance, some fibrosis mouse models are reversible, and collagen production may be part of the repair process. Given the large number of possible cell sources, future studies will have to use modern molecular techniques, such as fate-mapping, to create strategies to reverse skeletal muscle fibrosis.

Chronic activation of PDGFRα results in widespread organ fibrosis (Olson and Soriano, 2009), indicating that PDGFRα + cells may have a role in skeletal muscle fibrosis. Type-1 pericytes and FAPs express this receptor (Joe et al., 2010; Uezumi et al., 2010, 2011; Birbrair et al., 2013a), and like pericytes, FAPs line the skeletal muscle vasculature (Joe et al., 2010), suggesting their roles may overlap (Birbrair et al., 2013a). The extent of perivascular PDGFRα+ cells' contribution to skeletal muscle fibrosis has not been demonstrated. Future studies should use fate-mapping of endogenous skeletal muscle PDGFRα+ cells exposed to distinct conditions leading to fibrosis. Determining whether PDGFRα+ pericytes and PDGFRα+ FAPs are lineage-related and whether their roles in skeletal muscle fibrosis vary would also be interesting.

As with PDGFRα, the selective overexpression of a disintegrin and metalloprotease 12 (ADAM12) in the skeletal muscle increases fibrosis and suppresses regeneration (Jorgensen et al., 2007). In an elegant study, researchers used an inducible, tetracycline-dependent, cell-fate mapping system. They generated triple transgenic mice that expressed tetracycline transactivator under control of the ADAM12 locus, the conditional reporter RosaYFP, and the recombinase Cre under control of the tetracycline transactivator. Labeling the cells derived from ADAM12+ cells was temporally controlled by administering doxycycline, which prevents Cre expression, and they inducibled genetic ablation of ADAM12+ cells in the skeletal muscle. Their findings revealed that pericytes expressing ADAM12 during development, located in very close proximity to blood vessel endothelial cells, give rise to most of the collagen-producing cells during skeletal muscle injury (Dulauroy et al., 2012). However, fibrosis formation was analyzed in healthy, young skeletal muscle after injury. Future studies should explore whether the collagen-producing cells in the skeletal muscle of mdx and old mice have the same ancestors.

A new finding complements the evidence that pericytes are the source of collagen-producing cells in the fibrous tissue deposited in old skeletal muscle. Birbrair et al. (2013d) found that the pericytes involved in scar formation in the skeletal muscle of old mice differ from those associated with skeletal muscle regeneration; only type-1 pericytes contribute (Birbrair et al., 2013d). Future studies should use this pericyte subpopulation as a cellular target to reduce fibrosis in older mammals.

The cellular source of fibrosis in chronic diseases, such as Duchenne muscular dystrophy, remains unknown. To what extent type-1 pericytes contribute to collagen-producing cells in the skeletal muscle in comparison with other cell populations that give rise to those cells is also unclear. The detailed fate-mapping and lineage-tracing experiments that confirm pericyte participation in skeletal muscle fibrosis have not been done for other possible cellular sources of collagen-producing cells.

The basic molecular mechanisms involved in fibrous tissue deposition in the skeletal muscle are not completely understood. The well-studied cytokine transforming growth factor β (TGFβ), which is released from injured myofibers, seems to be essential to fibrous tissue formation (Massague, 2012). It binds to transmembrane receptor TGFβ receptor type II, recruiting TGFβ receptor type I to the complex. Both receptors have serine/threonine kinase activity and form heteromeric complexes in the presence of the activated ligand. TGFβ binding to the extracellular domains of type I and type II receptors initiates signaling cascades across the cell membrane by inducing transphosphorylation. It subsequently activates the type I receptor at the glycine/serine (GS)-rich domain, which acts as a phosphorylation site with receptor kinase activity (Kang et al., 2009). The type 1 receptor then catalyzes activation of the intracellular SMAD transcription factors (Massague et al., 2005), which stimulate transcription of specific target genes, leading to the production of extracellular matrix proteins and fibrosis formation (Lieber and Ward, 2013) that interfere with skeletal muscle regeneration and function (Gharaibeh et al., 2012). In contrast, inhibiting TGFβ reduces fibrosis and promotes muscle regeneration (Fukushima et al., 2001; Sato et al., 2003).

TGFβ is involved in a range of biological processes (Heldin et al., 2009; Padua and Massague, 2009; Hawinkels and Ten Dijke, 2011; Dooley and Ten Dijke, 2012; Pardali and Ten Dijke, 2012). Thus, detailed understanding of which cells respond to its signaling is required for the design of effective therapeutic approaches without undesirable side effects. At least *in vitro*, type-1 pericytes respond to TGFβ, increasing type I collagen production (Birbrair et al., 2013d), while type-2 did not seem to respond under the same conditions (Birbrair et al., 2013d). Future experiments should test whether TGFβ signaling is required for pericytes to participate in skeletal muscle scarring *in vivo*. One indication for this requirement is that TGFβ induces ADAM12 expression (Solomon et al., 2010; Dulauroy et al., 2012), which plays an important role in pericytes' fibrotic response.

Another member of the TGFβ protein superfamily, myostatin (McPherron et al., 1997), also known as GDF-8, not only controls skeletal muscle growth, but also regulates the progression of fibrosis (Li et al., 2008). Connective tissue growth factor (CTGF) is another molecule that has been shown to reproduce many of the profibrotic effects of TGFβ in skeletal muscle. Elevated levels of CTGF have been detected in skeletal muscle from mdx mice, dystrophic dogs, and patients with Duchenne muscular dystrophy (Sun et al., 2008; Vial et al., 2008). Whether pericytes express receptors and respond to myostatin and CTGF has yet to be explored. TGFβ can also induce production of PDGFs (Bonner, 2004). As pericytes express receptors to these ligands (Hellstrom et al., 1999), whether this signaling pathway plays a role in skeletal muscle fibrosis *in vivo* should be explored. Fibroblasts that express MMP9 and PlGF help to recover the vascular network structure by diminishing collagen deposition in the skeletal muscle of old dystrophic mice (Gargioli et al., 2008). Whether the expression of these factors differs between the two pericyte subtypes is unknown.

For full functional recovery of skeletal muscle affected by chronic diseases, aging, and trauma, fibrosis must be limited. Effective repair of skeletal muscle under these conditions cannot be achieved yet. More studies are needed to define the cellular and molecular mechanisms and functional significance of fibrosis in healthy, young and diseased, old skeletal muscles. Although pericytes play an important role in this process, detailed fate-mapping and lineage-tracing experiments would significantly advance the field.

## Pericyte contribution to fat accumulation in skeletal muscle

Accumulation of ectopic adipocytes in skeletal muscle is typical of such disorders as obesity, sarcopenia, and dystrophies and provides an accurate assessment of the severity of Duchenne muscular dystrophy (DMD) (Wren et al., 2008). Increased fat is also observed in the skeletal muscle of older adults (Goodpaster et al., 2004; Goodpaster and Wolf, 2004; Visser et al., 2005).

The origin of these fat cells has been revealed only recently. A group of cells in the perimysium, particularly the perivascular space (Greco et al., 2002), where fat accumulation is most evident, express platelet-derived growth factor receptor α (PDGFRα), the major contributor to ectopic fat cell formation in skeletal muscle. These cells are quiescent in intact muscle but proliferate efficiently in response to damage. PDGFRα+ cells differ from satellite cells and are located in the muscle interstitial space between myofibers, close to blood vessels (Joe et al., 2010; Rodeheffer, 2010; Uezumi et al., 2010). Skeletal muscle pericytes can differentiate *in vitro* toward adipogenic lineage (Farrington-Rock et al., 2004; Crisan et al., 2008a) but, like PDGFRα+ cells, do not generate myofibers, and only type-1 express PDGFRα. When purified type-1 pericytes are delivered intramuscularly in a mouse model of fatty infiltration, ectopic white fat is generated (Birbrair et al., 2013a). This approach clearly identifies their adipogenic potential, but only lineage-tracing will demonstrate that type-1 pericytes become fat cells in skeletal muscle *in vivo* under physiological conditions.

Whether perivascular PDGFRα + cells have a physiological role in the various illnesses characterized by muscular ectopic fat accumulation, such as myopathies and obesity, and whether modifying cell properties by manipulation and grafting would influence their fate *in vivo* are unclear. Although genetic tracing techniques were used to track these cells in other tissues (Lee and Granneman, 2012), confirming their capacity to become fat cells in skeletal muscle will require lineage-tracing studies. Testing whether depleting specific PDGFRα+ perivascular cells would prevent fat formation in skeletal muscle would also be interesting.

Is activating PDGFRα important for the adipogenetic role of PDGFRα + perivascular cells in skeletal muscle? Most of the primary functions of PDGFα and platelet-derived growth factor receptor α (PDGFRα), were unknown because Pdgfa and Pdgfra knockout mice die either as embryos or shortly after birth. Recent experiments using conditional gene ablation and gain-of-function transgenics (Gnessi et al., 1993; Bostrom et al., 1996; Soriano, 1997; Fruttiger et al., 1999; Karlsson et al., 1999, 2000) showed that PDGFα receptors are crucial for the proper development of several tissues (Crosby et al., 1998; Bostrom et al., 2002; Ostman, 2004). After ligand binding, the kinase domains of PDGFRα phosphorylate tyrosine residues of the receptor's cytoplasmic domain, which act as docking sites for phosphatidylinositol 3-kinase, STATs, SRC family kinases, SHP2 phosphatase, and phospholipase Cγ (Vignais and Gilman, 1999; Lakner et al., 2010; Xiong et al., 2010; Lin et al., 2014). These pathways regulate such transcription factors as SREBP, FOXO, c-MYC, and AP1, which are involved in cell growth, proliferation, differentiation, survival, and migration (Besancon et al., 1998; Tsatsanis and Spandidos, 2000; Guida et al., 2007; Erovic et al., 2012). They have also been linked to diseases characterized by fat accumulation in blood vessel walls, such as atherosclerosis (Tedgui and Mallat, 2006; Artwohl et al., 2009; Feinberg, 2013; Li et al., 2013). However, the function of PDGFα ligands and their receptors in skeletal muscle adipogenesis remains unclear. The pericyte marker neural/glial antigen 2 (NG2) proteoglycan (Ozerdem et al., 2001) binds to PDGFα (Goretzki et al., 2000) and may function as its co-receptor with a potential effect on the respective cell-surface signaling receptor (PDGFRα) (Grako and Stallcup, 1995; Grako et al., 1999). Future studies may determine whether the fate of PDGFRα + pericytes changes when they are exposed to PDGFα and whether their differentiation potential remains unchanged after exposure to PDGFRα-Fc chimeric receptors, which compete with their receptors for ligands *in vitro*. Loss-of-function and gain-of-function assays may demonstrate whether PDGFRα in pericytes regulates fat formation in skeletal muscle.

Systemic factors, such as hormone levels and nutrients, may play a role in regulating PDGFRα + cells' adipogenic potential. For instance, a high-glucose medium was reported to enhance adipogenic differentiation of skeletal muscle-derived cells (Aguiari et al., 2008), suggesting that the microenvironment may determine the fate of cells that sense changes in skeletal muscle physiology. The ability to target skeletal muscle PDGFRα + perivascular cells exclusively will open new therapeutic strategies for skeletal muscle diseases caused by, or associated with, severe adipose tissue accumulation.

Pericyte participation in fat infiltration of skeletal muscle has been confirmed (Birbrair et al., 2013a), providing a cellular target susceptible to pharmacological modulation and signaling manipulation. This strategy will require more detailed analyses.

## Pericytes and ectopic bone formation in skeletal muscle

Heterotopic ossification, the ectopic formation of bone and/or cartilage in soft tissues, such as skeletal muscles outside the periosteum, happens only in genetic disorders, such as fibrodysplasia ossificans progressiva and progressive osseous heteroplasia (Adegbite et al., 2008; Yu et al., 2008; Kaplan et al., 2012). In the skeletal muscles of mdx mice (dystrophin-deficient mouse model of Duchenne muscular dystrophy) (Kikkawa et al., 2009; Mu et al., 2013), this debilitating condition may be induced by the inflammation associated with trauma. However, other causes have been reported (Thorseth, 1968; Sirvanci et al., 2004; McCulloch and Bush-Joseph, 2006; Bek et al., 2009; Kim and Choi, 2009; Chouhan et al., 2012; Kalenderer et al., 2012).

The biological mechanism leading to osteoinduction in the skeletal muscle under physiological conditions has not been identified, and the exact cellular origin of heterotopic ossification is not well characterized. Nevertheless, recent sophisticated studies have made advances. The use of Cre/loxP technology allows investigators to track specific cell lineages (Liu et al., 2004; Maes et al., 2010). Several studies used murine models harboring real-time visual transgenes and Cre/loxP technology as a powerful way to identify which cells in skeletal muscle give rise to bone-forming cells (Kan et al., 2009, 2013; Lounev et al., 2009; Medici et al., 2010; Chakkalakal et al., 2012). Injury may provoke a local inflammatory reaction, and cytokines released into the blood might prompt circulating immune cells to differentiate into osteoblasts. However, using CD19-Cre, LCK-Cre, and Lyz-Cre transgenic mice, researchers have shown that B cells, T cells, and macrophages/monocytes, respectively, do not generate them (Kan et al., 2009). Somite-derived cells were excluded using Nestin-Cre reporter mice, and myoblasts, which are more committed to the myogenic lineage, were excluded using Myf5-Cre (Kan et al., 2009) and MyoD-Cre (Lounev et al., 2009) transgenic mice. These results are consistent with the fact that, during the generation of ectopic bone, the early immune response in skeletal muscle lesions kills myoblasts (Shore and Kaplan, 2010).

Recent investigations have suggested that cells residing in the skeletal muscle interstitial space contribute to some of the ectopic bone tissue (Wosczyna et al., 2012). However, their precise identity was not determined (Bosch et al., 2000). Histological analyses of heterotopic lesions from patients with fibrodysplasia ossificans progressiva demonstrate positive staining for endothelial markers, such as the angiopoietin receptor, Tie2, in ectopic chondrocytes and osteoblasts, suggesting a possible role for endothelial cells (Lounev et al., 2009; Medici et al., 2010). However, this marker is not specific to endothelial cells; in fact, it is also expressed in pericytes (Park et al., 2003; Cai et al., 2008). Cells expressing Tie2 receptor respond to inflammatory triggers, differentiate into osteogenic lineage, and contribute greatly to heterotopic bone in animal models of fibrodysplasia ossificans progressiva (Lounev et al., 2009; Wosczyna et al., 2012). Lineage-tracing studies using Tie2-Cre reporter mice have also pointed to these cells in generating the chondrocytes and osteoblasts found in skeletal muscle lesions (Lounev et al., 2009; Medici et al., 2010; Chakkalakal et al., 2012). Other analyses revealed that skeletal muscle osteogenic progenitors, distinct from satellite cells, express PDGFRα (Oishi et al., 2013). They undergo osteogenic differentiation both *in vitro* and *in vivo* in response to osteogenic conditions and/or BMP stimuli (Uezumi et al., 2010; Oishi et al., 2013) and have been observed surrounding ectopic bone tissues after trauma in humans. In skeletal muscle, PDGFRα + cells accumulate around blood vessels (Uezumi et al., 2014) and include type-1 pericytes (Birbrair et al., 2013a), suggesting that a pericyte subpopulation may also form ectopic bone in skeletal muscle. Blood vessels could be a source of osteogenic progenitor cells, which differentiate into osteoblasts, for example, when inflammatory cytokines are released by macrophages. However, this hypothesis has not been tested experimentally.

Skeletal muscle pericytes have chondrogenic potential *in vitro* (Crisan et al., 2008a), and Li et al. (2011) showed that cells residing in the skeletal muscle fascia have strong chondrogenic potential but lack pericyte marker CD146. Crisan et al. (2008b) also reported that vascular pericytes may differentiate into osteoblasts. Levy et al. (2001) and other groups suggested a similarity between osteoprogenitors in the skeletal muscle and pericytes isolated from intramuscular connective tissue (Diaz-Flores et al., 1992a; Gronthos and Simmons, 1996; Reilly et al., 1998; Kuznetsov et al., 2001; Levy et al., 2001; Crisan et al., 2008a). Whether they contribute to the heterotopic ossification that occurs in skeletal muscle *in vivo* is not known Although in bone marrow pericytes are capable of bone formation (Shi and Gronthos, 2003; Sacchetti et al., 2007; Mendez-Ferrer et al., 2010b), their characteristics may vary significantly between tissues (Armulik et al., 2011).

A recent lineage-tracing study using GLAST-CreER mice identified GLAST-expressing cells as precursors that contribute to heterotopic ossification (Kan et al., 2013). GLAST (glutamate aspartate transporter) is expressed in various central nervous system (CNS) cells, such as Muller, Bergmann glia, astrocyte, and neural stem cells (Danbolt et al., 1992; Levy et al., 1993; Lehre et al., 1995; Shibata et al., 1997; Izumi et al., 2002; Slezak et al., 2007; Ehm et al., 2010), but can also be found in other cell types, such as pericytes in the spinal cord (Goritz et al., 2011). Whether GLAST is expressed in cells outside the CNS is not known, and verifying whether skeletal muscle pericytes express GLAST would be especially interesting. Supporting this idea, most GLAST-creER labeled cells in skeletal muscle interstitium were closely associated with vasculature (Kan et al., 2013). This study did not specify the cellular origin, but approximately 35% of the ectopic bone-producing cells in the lesions clearly belong to a GLAST-expressing lineage. Is a specific pericyte subpopulation responsible? What is its contribution compared to other cell types'? The possibility that osteoprogenitor cells might originate from perivascular cells highlights the strong association between angiogenesis and the heterotypic ossification of skeletal muscle (Hegyi et al., 2003).

The basic molecular mechanisms involved in ectopic calcification in skeletal muscle are not known. Critical inductive factors and a permissive environment may affect specific cell types and contribute to heterotopic ossification (Chalmers et al., 1975; Baird and Kang, 2009). A recent study suggests that the same mechanism that induces vascular calcification gives rise to osteoprogenitor cells (Yao et al., 2013). Elucidating these mechanisms is important since we have no effective and safe therapy to prevent this condition. Regulatory molecules acting on the perivascular cells necessary for the development of traumatic heterotopic ossification should be further investigated.

The bone morphogenetic proteins (BMP) of ALK2 ligands, such as BMP2, BMP4, and BMP9, might be primary inducers of heterotopic ossification; mixed with Matrigel and injected into the skeletal muscles of mice, they have osteoblastic activity (Chen et al., 2012; Nishimura et al., 2012), and they are highly expressed in human lesions with heterotopic ossification (Gannon et al., 1997; Grenier et al., 2013). Transgenic mouse models of heterotopic ossification with specific signaling molecules, such as BMPs, deleted should be used in different cellular targets to verify whether those molecules are essential for *in vivo* ectopic bone formation in skeletal muscle in physiological conditions. Future studies should determine whether BMP receptors are expressed in pericytes. Additionally, the fate of pericyte subtypes exposed to BMPs should be investigated to determine whether their differentiation potential remains unchanged after exposure to BMPR-Fc chimeric receptors, which compete with pericyte receptors for ligands. Future efforts should focus on the activation of osteogenic potential by such less-studied molecules as the growth factor Nell-1, which induces osteogenic differentiation in pericytes (Zhang et al., 2011). The discovery of such signals and a better understanding of the exact role of pericytes in skeletal muscle ectopic calcification would support development of therapeutic strategies to treat this clinically significant condition.

## Conclusions

Pericytes play several critical roles in skeletal muscle repair, and elucidating how their tissue- formation capabilities contribute to skeletal muscle pathophysiology will be important to future treatments. Based on their molecular markers and specific functions, muscular pericytes have been identified as heterogeneous, and at least two subpopulations have been described. Taking their diversity into account, information regarding pericytes will be crucial in advancing our understanding of skeletal muscle disease and aging.

### Conflict of interest statement

The authors declare that the research was conducted in the absence of any commercial or financial relationships that could be construed as a potential conflict of interest.
